# Unlocking the potential of engineered circular RNA therapeutics

**DOI:** 10.1007/s44258-026-00079-5

**Published:** 2026-03-19

**Authors:** Xueyan Zhen, Mahyar Mahmoudi, Xinrui Lan, Guanheng Huang, Wei Tao

**Affiliations:** Center for Nanomedicine, Department of Anesthesiology, Perioperative, and Pain Medicine, Brigham and Women’s Hospital, Harvard Medical School, Boston, MA 02115 USA

**Keywords:** Circular RNA, RNA therapeutics, Stability, Immunogenicity, RNA vaccine

## Abstract

Linear RNA-based therapeutics such as messenger RNAs, antisense oligonucleotides, and small interfering RNAs are limited by susceptibility to nuclease degradation, reduced in vivo stability, and potential immunogenicity, which hinder their broader clinical applications. Circular RNAs (circRNAs) represent a type of single-stranded RNA molecule characterized by a covalently closed circular structure formed through back-splicing. Due to their resistance to exonuclease-mediated degradation and relatively decreased innate immune activation, circRNAs represent a versatile RNA tool capable of long-lasting protein expression. Endogenous circRNAs regulate gene expression via miRNA sponging and protein interactions and, in select cases, support cap-independent translation. Dysregulated circRNAs have been implicated in cancer, metabolic disease, and inflammation. Recent technological advances have enabled efficient engineering of synthetic circRNAs, incorporating optimized components for enhanced circularization, purification, and translational performance. In addition, progress in delivery platforms, including lipid nanoparticles, viral vectors, and biomimetic carriers, has expanded the therapeutic potential of circRNAs across oncology, infectious diseases, and immune modulation. Here, we summarize the biological features and therapeutic applications of circRNAs, highlight strategies to overcome manufacturing and delivery challenges, and discuss the future opportunities for unlocking the clinical potential of engineered circRNA therapeutics.

**Graphical Abstract:**

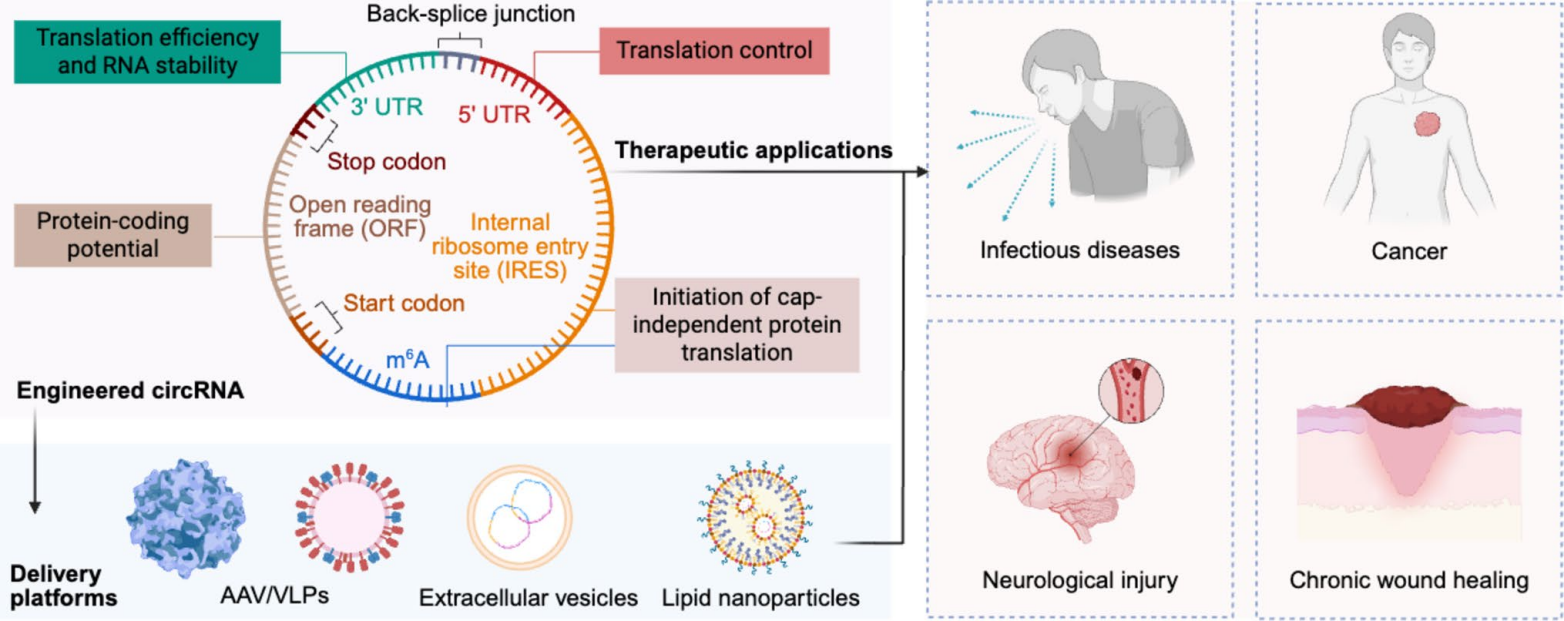

## Highlights

- Covalently closed circular RNAs provide nuclease resistance and low immunogenicity with diverse biological functions.
- Insights cover design, manufacturing strategies, and translation to achieve high-level and long-lasting protein output.
- This review summarizes advances and hurdles in circular RNA delivery platforms and therapeutic applications.

## Introduction

In recent years, the significant success of messenger RNA (mRNA) vaccines in combating Coronavirus Disease 2019 (COVID-19) has marked a transformative moment in the field of RNA therapeutics [1–5], especially in highlighting the clinical potential of nucleic acid-based drugs. RNA-based therapeutic strategies have subsequently gained recognition as flexible and programmable modalities due to their scalability and adaptability for personalized medicine. To date, several classes of linear RNA-based therapeutics, including antisense oligonucleotides (ASOs), small interfering RNAs (siRNAs), microRNAs (miRNAs), aptamers [6], and therapeutic mRNAs [7, 8], have gained FDA approval for clinical applications [9]. These breakthroughs, which facilitated a timely and effective response to a worldwide public health emergency, not only demonstrated the potential of RNA-relevant platforms, but also laid the foundation for expanding RNA modalities. However, despite these advances, linear RNAs face several intrinsic limitations that constrain their broader clinical use, such as vulnerability to degradation by endogenous and environmental ribonucleases (RNases) [10–12], reduced stability during storage and in vivo, and the risk of eliciting strong immune responses. These challenges have prompted growing interest in an alternative RNA class, circular RNAs (circRNAs), as the next-generation therapeutic agents with improved stability [13–15], reduced immunogenicity, and increased translational potential [16].

Unlike their linear RNA counterparts, circRNAs possess a covalently closed, single-stranded circular structure. They are formed via a noncanonical back-splicing mechanism [17–21], in which a downstream splice donor is ligated to an upstream splice acceptor, thereby eliminating the free 5’ and 3’ ends that are typically susceptible to exonuclease attack. As a consequence of this closed-loop architecture [13, 19, 22–24], circRNAs display markedly enhanced structural stability and exhibit strong protection against RNase-driven degradation, leading to substantially extended molecular persistence compared with linear RNAs. Advances in high-throughput sequencing technologies [25–27], together with computational approaches [28–30], have revealed that circRNAs are not merely splicing by-products, but rather abundant and conserved molecules with diverse biological functions. Consistent with these properties, circRNAs in mammalian systems often demonstrate half-lives exceeding those of their linear mRNA counterparts by more than 2.5-fold, underscoring their intrinsic advantages for therapeutic development.

For a long time, the biological functions of circRNAs remained unknown [31]. Recent evidence now demonstrates that circRNAs participate in diverse layers of gene regulatory processes. These functions encompass sequestration of miRNAs [13, 32–34], modulation of mRNA translation [35, 36], interactions with RNA-binding proteins (RBPs) [37–39], and, in certain contexts, the production of bioactive peptides via internal ribosome entry site (IRES)-dependent mechanisms [40–44] or N6-methyladenosine (m^6^A)-mediated translation [45, 46]. In this review, we present an integrated overview of circRNA synthesis, structural properties, and therapeutic relevance. We first outline the molecular characteristics of endogenous circRNAs, including their covalently closed topology, enhanced stability, and versatile regulatory functions such as miRNA sponging, protein interactions, and cap-independent translation. We then discuss the recent advances in the design and production of engineered circRNAs, highlighting strategies for efficient circularization, purification, and optimization of translational elements to achieve durable and high-level protein expression. We further examine delivery platforms for circRNA therapeutics, including lipid nanoparticles (LNPs), virus-derived carriers, and biomimetic vectors. In addition to different therapeutic applications, we highlight the emerging utility of circRNAs as biomarkers due to their stability and detectability in biofluids. We then describe the key challenges that remain for clinical translation of circRNAs, including large-scale manufacturing, removal of linear RNA contaminants, enhancement of translational efficiency, and tissue-specific delivery. Finally, we identify future directions for translating circRNA technologies into clinical practice.

## Synthesis and features of engineered circRNA therapeutics

### Endogenous circRNAs

**Fig. 1:**
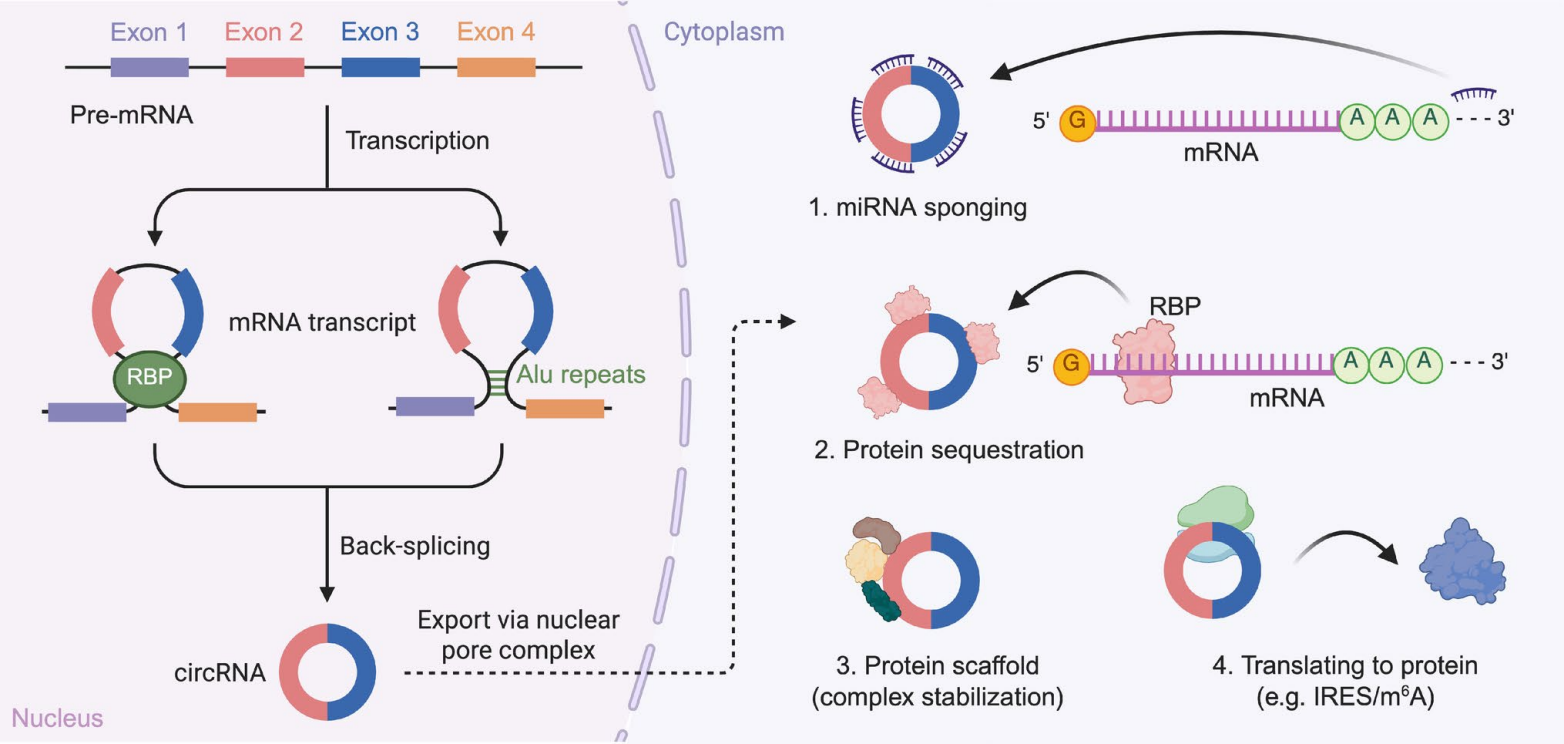
Biogenesis and functional mechanisms of circRNAs. CircRNAs are generated through a noncanonical back-splicing event, giving rise to a covalently closed circular topology that is resistant to exonuclease degradation. This process can be promoted by RBPs or inverted repeat elements (such as Alu repeats). Once exported to the cytoplasm, circRNAs exert diverse regulatory functions: (1) acting as miRNA sponges to modulate target gene expression; (2) sequestering RBPs and thereby preventing their interaction with linear transcripts; (3) serving as protein scaffolds to stabilize multiprotein complexes; and (4) in some cases, functioning as templates for cap-independent translation through IRES or m^6^A modifications, generating small functional peptides

Endogenous circRNAs are primarily generated through a noncanonical splicing event known as back-splicing, wherein a downstream 5’ splice donor is covalently joined to an upstream 3’ splice acceptor [47–50]. This process forms a closed-loop RNA molecule through a covalent 3’−5’ phosphodiester linkage at the back-splice junction [51]. In contrast to linear splicing, which produces open-ended transcripts with defined 5’ and 3’ terminals, back-splicing generates a covalently closed structure without free ends, making circRNAs resistant to exonuclease-mediated degradation [52–54]. As a result, circRNAs often display greater stability and longer half-lives compared to their linear counterparts. Functionally, endogenous circRNAs participate in numerous post-transcriptional regulatory processes. Many act as molecular sponges that sequester miRNAs [55], thereby modulating the expression of their target genes. Others interact with RBPs [56–58], influencing their localization, activity, or availability. In addition, a subset of circRNAs has been reported to contain IRES or m^6^A modifications that enable cap-independent translation [40, 42, 50] in some cases and give rise to functional peptides (Fig. 1).

### Production of engineered circRNAs in vitro

Unlike linear mRNA molecules, the generation of engineered circRNAs requires additional processing steps for circularization and purification, which must be carefully optimized to achieve high product quality, functional integrity, and low immunostimulatory activity. In vitro circularization typically involves ligating the 3’-OH and 5’-phosphate terminals of in vitro-transcribed linear RNA substrates to form a covalently closed loop structure via a 3’−5’ phosphodiester linkage.

**Fig. 2:**
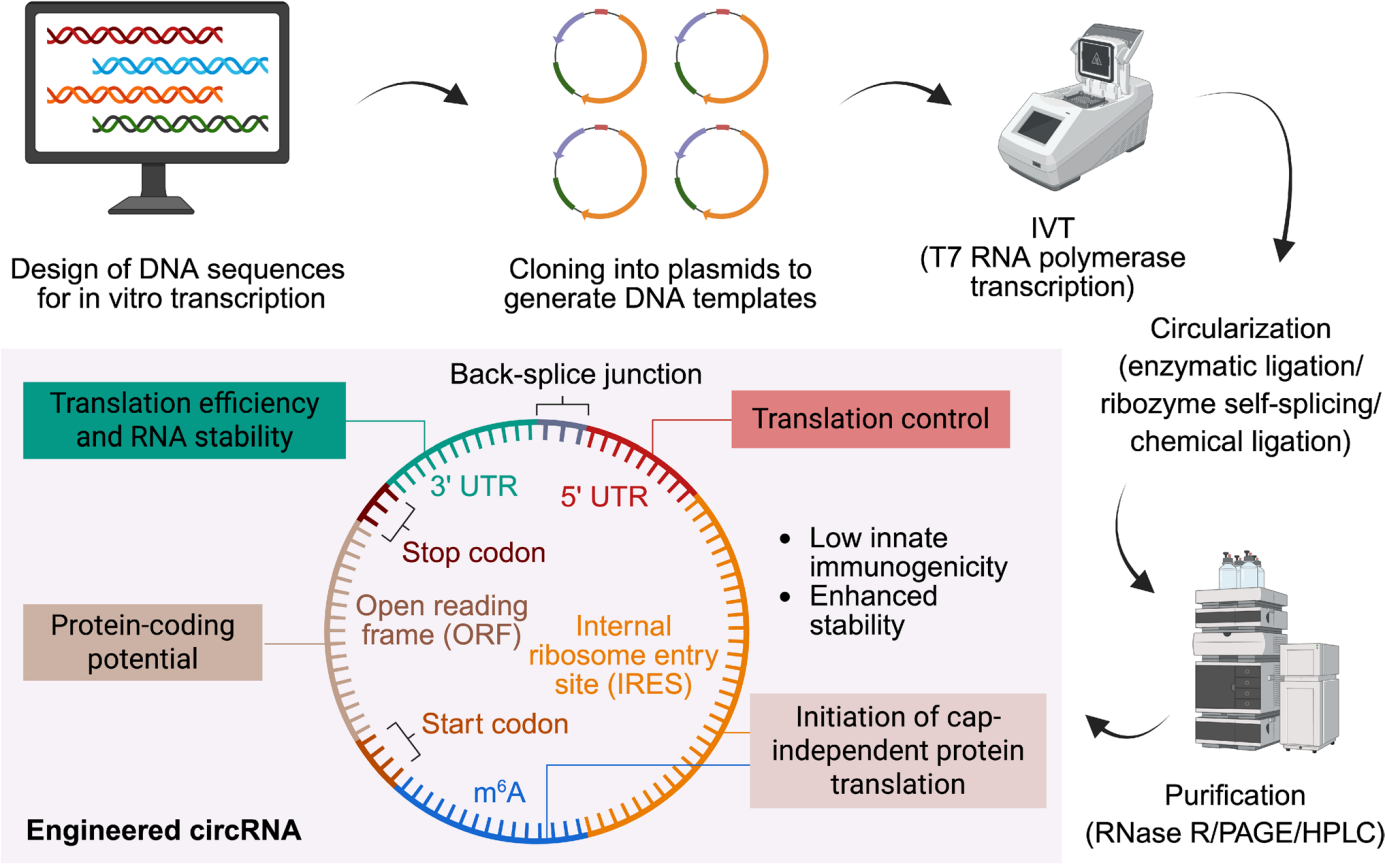
In vitro synthesis and design principles of engineered circRNAs. Schematic illustration of the workflow for generating engineered circRNAs in vitro. First, DNA sequences are designed on the basis of the desired open reading frame (ORF) and regulatory elements. These DNA constructs are cloned into plasmids to produce templates for transcription. Linear RNA precursors are then synthesized by in vitro transcription (IVT) using T7 RNA polymerase, followed by circularization via enzymatic ligation, ribozyme-mediated self-splicing, or chemical ligation. Rigorous purification procedures, including RNase R digestion, PAGE, or HPLC, are applied to remove linear RNA contaminants and enrich high-purity circRNAs. The resulting engineered circRNAs contain regulatory features such as untranslated regions (UTRs), start and stop codons, back-splice junctions, and cis-elements (IRES or m^6^A) that govern RNA stability, translation efficiency, protein-coding potential, and translation control

Multiple approaches exist to circularize RNA, including chemical ligation [35, 49, 59, 60], enzymatic ligation [60, 61] (e.g., using T4 RNA ligases [61, 62]), and ribozyme-mediated self-splicing [59]. Regardless of the method employed, circularization is often accompanied by the accumulation of residual linear RNA precursors, which can trigger innate immune responses through recognition by cytosolic RNA sensors. Therefore, rigorous purification of the circularized product is essential to improve the therapeutic potential of engineered circRNAs [50, 63, 64]. Two primary approaches are commonly used to enrich and purify circRNAs. The first strategy utilizes RNase R, a 3′−5′ exoribonuclease that preferentially degrades linear RNA species while leaving circular RNA structures largely intact [30, 65]. However, prolonged exposure to RNase R may inadvertently introduce nicks into the circRNA, thereby compromising structural integrity and product purity. The second approach relies on size-based purification methods, such as polyacrylamide gel electrophoresis (PAGE) [65, 66] and high-performance liquid chromatography (HPLC) [59, 63], which enable separation of circRNAs based on molecular size. These techniques offer high selectivity and sensitivity, and have been reported to generate circRNA preparations with purity exceeding 90% in scalable production settings. Effective removal of linear contaminants following purification not only enhances product quality but also reduces the possibility of activating RNA-sensing pathways, thereby mitigating immunogenicity and improving the translational performance of synthetic circRNA therapeutics (Fig. 2).

### Properties of therapeutic circRNAs

circRNAs possess unique molecular features that endow them with significant therapeutic potential, including high stability and resistance to exonucleolytic degradation. Their covalently closed structure leads to extended half-lives compared with linear RNAs, making them attractive candidates for applications that require durable expression. In addition, IVT circRNAs, when appropriately purified, display reduced innate immunogenicity relative to linear mRNAs [53, 63, 67–69], which can be advantageous in certain therapeutic contexts.

#### Enhanced protein production and intracellular stability

Circularization has the capacity to reshape the pharmacological properties of RNA therapeutics by prolonging the functional lifespan of molecules that would otherwise be short-lived. Significant progress has been made in synthesizing long transcripts and engineering their efficient circularization. However, because circRNAs lack the 7-methylguanosine (m^7^G) cap [70] characteristic of linear mRNAs, well-established principles for enhancing mRNA translation cannot always be directly applied to circRNAs. Instead, circRNA translation relies on alternative regulatory elements. Systematic studies have identified several key features that govern circRNA translation efficiency, including vector topology [71], UTR design [41], IRESs [72], m^6^A motifs [73, 74], and synthetic aptamers. Optimization of these elements has been shown to increase protein output from circRNAs by several 100-fold, enabling robust and durable expression in vivo. Combining these design principles, engineered circRNAs can outperform mRNAs in protein production in vitro while maintaining prolonged translation activity in vivo [13].

In addition to translation, the intrinsic stability of circRNAs further enhances their therapeutic value. Their covalently closed circular architecture renders them markedly more resistant to degradation than linear RNA counterparts. Recent advances in structural and chemical engineering have coupled stability with improved translatability, for example by introducing cap-mimicking features while preserving circular integrity. In one study, such multidimensional engineering enhanced in vivo protein expression by nearly an order of magnitude, resulting in markedly increased antibody titers, which were 17-fold higher after prime immunization and 3.7-fold higher after boosting in a severe acute respiratory syndrome coronavirus 2 (SARS-CoV-2) vaccine model [35]. In addition to large circRNAs, synthetic small circRNAs, which are typically shorter than 300 nucleotides, have been designed to encode one or multiple peptide antigens through site-specific ligation of RNA oligonucleotides. Incorporation of fluorogenic aptamers has facilitated real-time monitoring of their stability. Notably, small circRNAs exhibited exceptional thermal robustness, with solution storage half-lives approaching 400 days at −20 °C, and exhibited greater stability not only than small linear RNAs but also than chemically-modified mRNAs and large circRNAs. CircRNA sequencing further confirmed the nucleotide authenticity of small circRNAs after cellular transfection. These small circRNAs supported sustained peptide expression for at least 7 days in cells and, when formulated as vaccines, activated the retinoic acid-inducible gene I (RIG-I) signaling in dendritic cells, highlighting their dual potential for stable protein expression and innate immune stimulation in immunotherapy applications [75].

#### Immunogenicity

The immunogenic profile of circRNAs is shaped by both their intrinsic features and the quality of their production process. The innate immune system detects foreign RNA through pattern recognition receptors (PRRs), notably Toll-like receptors (TLRs) and RIG-I-like receptors (RLRs). IVT circRNAs can activate RIG-I [76, 77] and induce immune gene expression [78], particularly when unmodified or contaminated with linear RNA byproducts. Chemical modifications, including m^6^A, have been shown to attenuate the immunogenic properties of circRNAs, suggesting that unmodified transcripts retain some intrinsic ability to trigger innate responses. Importantly, studies have demonstrated that much of the observed immune activation originates from residual linear or double-stranded RNA impurities generated during synthesis, whereas highly purified circRNAs elicit minimal RIG-I activation and display intrinsically low immunogenicity. This distinction highlights that innate immune stimulation is largely impurity-driven rather than circRNA-inherent, demonstrating the critical importance of strict purification and quality control standards in the preparation of therapeutic circRNAs. Therefore, a clearer understanding of circRNA immunogenicity will be critical for guiding synthesis and purification strategies, selecting suitable therapeutic applications, and ultimately maximizing clinical benefit while streamlining manufacturing.

### Comparisons between circRNAs and mRNAs

Several key distinctions have been noted between linear mRNA [79–83] and circRNA therapeutics in the context of vaccines and biologics. CircRNAs are covalently closed loops lacking the 5’ cap and 3’ poly(A) tail of mRNA, a structural feature that makes circRNA far more resistant to exonuclease degradation, and thus more stable in biological and storage conditions. Recent studies have shown that certain circRNA vaccines can maintain structural integrity for months at 4 °C and remain stable after multiple freeze–thaw cycles, supporting cold-chain-friendly distribution in resource-limited settings. In practice, circRNA vaccines have demonstrated high stability (e.g., maintaining integrity for months at 4 °C and tolerating multiple freeze–thaw cycles [22]), whereas mRNA vaccines are comparatively unstable and generally require ultracold, RNase-free storage conditions to prevent rapid degradation. Additionally, unmodified mRNA is inherently highly immunostimulatory, activating innate immune sensors and contributing to reactogenic side effects. By contrast, highly purified IVT circRNAs can show minimal innate immune activation, but immunogenicity varies with sequence/design (e.g., exogenous elements) and manufacturing impurities [22, 84–86]. This means that mRNA vaccines typically need nucleotide modifications to mitigate undesired innate immune activation, while circRNA can achieve an even lower immunogenic profile naturally (with chemical modifications to circRNA further reducing any residual immunogenicity). Furthermore, circRNA therapeutics can sustain antigen expression for a longer duration and with greater cumulative output. Owing to their increased molecular longevity, circRNAs persist in cells and continue producing antigen for extended periods, prolonging antigen presentation in antigen-presenting cells [87, 88], whereas linear mRNA’s protein expression is limited by its rapid degradation. This prolonged protein translation from circRNA can translate into more durable immune responses, and in some comparisons circRNA constructs have yielded higher total antigen levels and neutralizing antibody titers than equivalent mRNA constructs.

Regarding translation mechanisms, there is also a fundamental difference in protein translation between the two platforms. mRNA relies on the 5’ cap-dependent translation initiation mechanism, whereas circRNA lacks a cap and instead utilizes IRES elements for cap-independent translation. Advances in circRNA design (e.g., optimized IRES and UTRs) have improved the translation efficiency of circRNA vaccines to levels comparable to conventional mRNA, while benefiting from the circRNA’s sustained translational activity. From a manufacturing perspective, circRNA vaccines offer simplicity in synthesis because they eliminate the need for 5’ capping and polyadenylation. However, they are typically circularized via ribozyme-mediated self-splicing (or enzymatic ligation), and purification remains a complex, unsolved challenge due to the difficulty in removing similar impurities. In contrast, mRNA vaccine production involves additional design elements (cap structures, poly(A) tails, optimized UTRs) and rigorous purification to remove dsRNA contaminants, and the extreme instability of mRNA necessitates costly cold-chain logistics. These considerations highlight circRNA as an emerging RNA therapeutic strategy, combining greater stability, lower unintended immunogenicity, prolonged antigen production, and more flexible handling, while effectively harnessing the host translation machinery to induce potent immune responses.

## Delivery of circRNA-based therapeutics

**Fig. 3:**
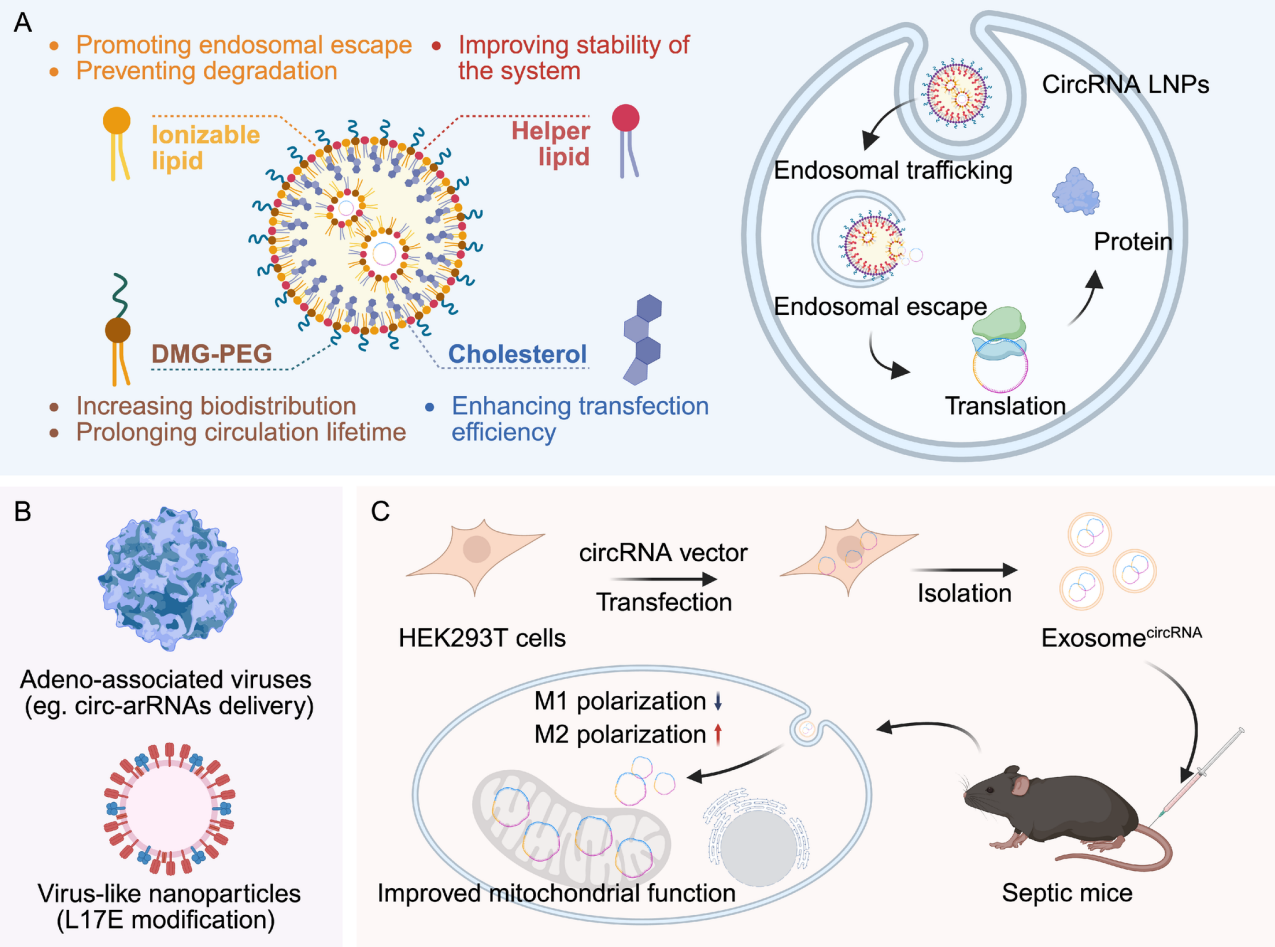
Delivery strategies for circRNA-based therapeutics. **A** LNP-based delivery of circRNAs. LNPs typically consist of ionizable lipids, helper lipids, cholesterol, and PEGylated lipids (e.g., DMG-PEG). Ionizable lipids promote endosomal escape and protect circRNAs from degradation; helper lipids enhance particle stability; cholesterol improves membrane fusion and transfection efficiency; and PEGylated lipids increase systemic stability and circulation time. After cellular uptake, circRNA LNPs undergo endosomal trafficking, escape into the cytoplasm, and subsequently support protein translation. **B** Virus-based delivery systems. AAVs have been adapted for circRNA delivery, such as in circ-arRNA therapeutic RNA editing. VLNPs, engineered with fusogenic peptide modifications (e.g., L17E), mimic viral morphology and enable efficient circRNA loading, membrane penetration, and endosomal escape. **C **Biomimetic delivery via exosomes. HEK293T cells can be transfected with circRNA vectors to produce circRNA-loaded exosome formulations. Isolated exosomes efficiently deliver circRNAs to target cells, where they modulate biological functions. For instance, circRNA-loaded exosomes have been shown to promote M2 macrophage polarization, improve mitochondrial function, and attenuate inflammation in septic mouse models [97]

Even when purified to homogeneity, therapeutic circRNAs still require an efficient delivery system to protect them from residual immune recognition, facilitate cellular uptake, and ensure expression at the intended site of action. Current strategies largely build upon the viral and non-viral platforms established for mRNA and siRNA delivery, while the unique circular topology of circRNAs may confer additional benefits in terms of stability and subcellular trafficking. In this section, we will highlight the major classes of circRNA delivery systems and their therapeutic applications (Fig. 3).

### Lipid-based delivery

LNPs have become the most established non-viral carriers for RNA therapeutics [89], for example, as demonstrated in mRNA vaccines. Their versatility and tunability also make them leading candidates for circRNA delivery. The lipid composition of LNPs critically determines encapsulation efficiency [90], biodistribution, protein expression, and the immunological outcomes of circRNA-loaded formulations.

Optimization of lipid components has been systematically investigated. Screening of ionizable lipids, helper lipids, and sterol analogues revealed strong influences on the physicochemical properties of circRNA LNPs, dendritic cell transfection in vitro, lymph node expression in vivo, and subsequent antigen-specific T cell responses [91]. In cancer immunotherapy, high-throughput combinatorial synthesis identified H1L1A1B3 as a potent formulation that enhanced circRNA transfection fourfold in lung cancer cells compared with the benchmark ALC-0315. When loaded with circRNAs encoding interleukin-12 (IL-12), H1L1A1B3 triggered robust antitumor immunity and tumor regression following intratumoral injection in Lewis lung carcinoma models [92]. Beyond oncology, circRNA LNPs have been engineered for tissue-specific delivery. For example, circSCMH1@LNP1 enabled nose-to-brain administration, achieving promising accumulation in peri-infarct regions after ischemic stroke. This delivery improved functional recovery by enhancing synaptic plasticity, vascular remodeling, and myelin repair [93]. In regenerative medicine, vascular endothelial growth factor A (VEGF-A) circRNAs produced via group I intron autocatalysis were encapsulated into U-105-derived LNPs using microfluidics. This formulation sustained VEGF-A expression and accelerated wound healing in diabetic ulcer models [94]. The adaptability of LNPs also extends to designs of oncolytic circRNAs. One example exploited human rhinovirus type 2 (HRV2) IRESs to restrict translation to cancer cells overexpressing EIF4G2 and PTBP1. Circularization using the permuted intron–exon (PIE) strategy and subsequent LNP encapsulation lowered immunogenicity and enabled efficient in vivo delivery. The resulting HRV2 IRES-driven GSDMD circRNA suppressed xenograft growth across multiple adenocarcinoma models [64]. Furthermore, LNPs can also be integrated with biomaterial platforms to enable localized and controlled release. In osteoarthritis therapy, a circRNA encoding a P65 super-repressor (srIκBα) was encapsulated in modified LNPs and incorporated into a silk fibroin–based composite hydrogel functionalized with MMP-responsive linkers. The resulting circ-srIκBα@LNP-SHC system provided environment-responsive release, attenuated NF-κB signaling, reduced inflammatory factor secretion, preserved extracellular matrix integrity, and slowed osteoarthritis progression [95].

Compared with other nanocarriers, LNPs combine efficiency with manufacturability. They can be produced at scale at relatively low cost, are compatible with high-throughput formulation screening, and allow precise modulation of physicochemical traits such as stability, pKa, and clearance rate. Microfluidic preparation enables highly uniform size distributions and encapsulation efficiencies exceeding 95%, while also facilitating reproducibility and quality control. Therefore, LNPs remain the most advanced and versatile platform for circRNA delivery.

### Virus-based delivery of circRNAs

Viruses have long inspired the design of delivery vehicles due to their natural efficiency in cellular uptake, endosomal escape, and protein expression. Building on this concept, virus-like nanoparticles (VLNPs) and adeno-associated viruses (AAVs) have been developed as promising carriers for circRNAs, combining biological efficiency with engineered safety.

VLNPs are self-assembled protein nanostructures that mimic the morphology and robustness of authentic viruses but lack viral genomes, thereby eliminating infectivity. Their inherent stability, tolerance to enzymatic degradation, and modular functionalization capacity make them attractive circRNA carriers. A representative example comes from papillomavirus-derived VLNPs, which were functionalized with a fusogenic peptide (L17E) on their surface. This modification markedly improved both membrane translocation and intracellular endosomal release of encapsulated circRNAs. In validation studies across different cell types, VLNP-L17E formulations enhanced target protein expression by approximately 1.5- to twofold compared with unmodified VLNPs [96].

In parallel, AAVs have been employed for circRNA delivery in therapeutic RNA editing. One notable approach involved engineered circular arRNAs (circ-arRNAs) designed to minimize unintended base-pairing, thereby substantially reducing off-target adenosine editing. In cell culture, circ-arRNAs improved both the efficiency and accuracy of editing of endogenous transcripts such as CTNNB1 and mutant TP53. When delivered via AAV in a mouse model of Hurler syndrome, circ-arRNAs repaired the disease-causing point mutation, restored α-L-iduronidase enzymatic activity, and lowered glycosaminoglycan accumulation in the liver [14]. This work, further advanced under the LEAPER 2.0 framework, demonstrates how virus-based circRNA delivery can expand therapeutic RNA editing with greater precision and broad applicability.

### Biomimetic vectors for circRNA delivery

Biomimetic carriers represent a conceptual framework encompassing both naturally derived and artificially engineered vesicle-like systems that mimic biological functions. Among them, extracellular vesicles (EVs) constitute a major category of natural biomimetic vectors, within which exosomes represent a specific subtype originating from the endosomal pathway. EVs and their subtype exosomes have gained particular attention for circRNA delivery, largely due to their intrinsic ability to evade immune detection and facilitate intercellular communication. Their endogenous origin endows inherent advantages, including low toxicity, high biocompatibility, and minimal immunogenicity, making them particularly attractive for in vivo applications.

Exosomes display surface complement regulatory proteins such as CD47, CD55, and CD59, which enable them to bypass immune clearance. Leveraging this property, researchers have encapsulated the murine homologue of circRNA SCAR into exosomes, as levels of this circRNA are markedly reduced in macrophages during sepsis, driving excessive M1 polarization. To enhance mitochondrial targeting, these exosomes were further electroporated with poly-D-lysine-graft-triphenylphosphine (TPP-PDL). Upon uptake by recipient cells, the engineered exosomes preferentially localized circRNA SCAR to macrophage mitochondria, thereby promoting M2 polarization and attenuating septic inflammation in vivo [97].

EVs, naturally secreted by virtually all cell types, range from 30 to 200 nm in size and play central roles in cell-to-cell signaling through the transfer of proteins, nucleic acids, and metabolites. Their intrinsic properties, such as low immunogenicity, high stability, and biocompatibility, render them well suited for therapeutic circRNA delivery. Based on these features, researchers employed a DNA construct containing introns from the HIPK3 gene to drive efficient back-splicing of split GFP and Cre recombinase exons, markedly enhancing circRNA biogenesis and packaging into EVs. Functionally, EVs carrying GFP circRNAs successfully mediated protein expression in HeLa cells. Furthermore, intravenous injection of EVs loaded with Cre recombinase circRNAs into Cre-LoxP reporter mice confirmed in vivo gene-editing capability, underscoring the therapeutic potential of this strategy [98]. By combining endogenous immune-evasion mechanisms with engineered enhancements such as organelle targeting or efficient back-splicing, biomimetic vectors open new avenues for harnessing circRNAs in therapeutic settings.

### Other delivery strategies

Beyond conventional nanoparticle- and vesicle-based systems, naked circRNAs and synthetic carriers such as charge-altering releasable transporters (CARTs) have also been explored for therapeutic delivery. Naked circRNAs, when administered directly, can trigger innate immune recognition, thereby providing insights into their intrinsic immunostimulatory capacity. Building on this, CARTs have emerged as versatile vehicles capable of encapsulating circRNAs and modulating their immunological profile. Recent studies have systematically evaluated the adjuvant-like properties of circRNAs by comparing T cell responses elicited by naked versus CART-delivered constructs, benchmarked against established vaccine adjuvants. Strikingly, immunization with CART-formulated circRNAs encoding tumor-associated antigens not only facilitated efficient antigen presentation but also induced potent cellular immune responses. In preclinical cancer vaccine models, this approach was sufficient to achieve robust tumor clearance, underscoring the translational potential of CART-circRNA systems [53].

## Engineered circRNA-based therapeutic applications

The rapid development of RNA medicines, accelerated by the COVID-19 pandemic, has led to the approval of mRNA vaccines and aroused interest in the next-generation platforms. CircRNA-based approaches are now being explored across viral infections, cancer [99–101], neurological injury, and metabolic diseases. In this section, we will outline their translational potential as both preventive and therapeutic agents.

### Cancer

Over the past decade, circRNAs have been regarded as a distinct class of regulatory molecules with diverse implications in cancer biology. Unlike their linear counterparts, circRNAs not only share similar binding partners but also expand the spectrum of gene regulation by acting as competitive inhibitors of miRNAs, scaffolds for protein interactions, or even templates for protein translation.

Several studies illustrate their roles in tumor progression and therapy resistance. In non-small cell lung cancer, for example, tumor-derived exosomal circRNA_102481 is enriched in patients with EGFR-TKI resistance, where it sequesters miR-30a-5p to restore ROR1 expression, ultimately promoting tumor growth and metastasis [102]. In KRAS-mutant tumors, circATXN7 directly binds the p65 subunit of NF-κB, blocking its nuclear localization and promoting immune evasion by making tumor-specific T cells resistant to activation-induced cell death [103]. Likewise, circASH2 is identified as a tumor-suppressive circRNA in aggressive hepatocellular carcinoma, where it enhances YBX1 phase separation and recruits hnRNP proteins to degrade TPM4 transcripts, thereby restraining metastatic dissemination [104]. Notably, although traditionally classified as non-coding, recent work has shown that subsets of circRNAs are indeed translatable, further broadening their functional repertoire [13].

These mechanistic insights are now being translated into therapeutic strategies using engineered circRNAs. One example comes from a high-throughput screen of ionizable LNPs, where the lead formulation H1L1A1B3 achieved a fourfold gain in transfection efficiency relative to ALC-0315 and, when loaded with IL-12 circRNA, elicited robust intratumoral immune activation and tumor regression in lung cancer models [92]. A different design utilized PIE splicing to circularize HRV2-IRES-driven constructs, which when encapsulated in LNPs, selectively suppressed EIF4G2^+^/PTBP1^+^ adenocarcinomas and even prevented tumor onset in KRASG12D-driven models by inducing persistent antigen-specific cytotoxic T cell responses [64]. Beyond immune modulation, circRNAs can also reprogram metabolism: circPETH encodes a peptide (circPETH-147aa) that enhances glycolysis in cancer cells. It simultaneously stabilizes SLC43A2 transcripts in T cells, causing amino acid deprivation and impairing cytotoxic lymphocyte function [105].

**Fig. 4:**
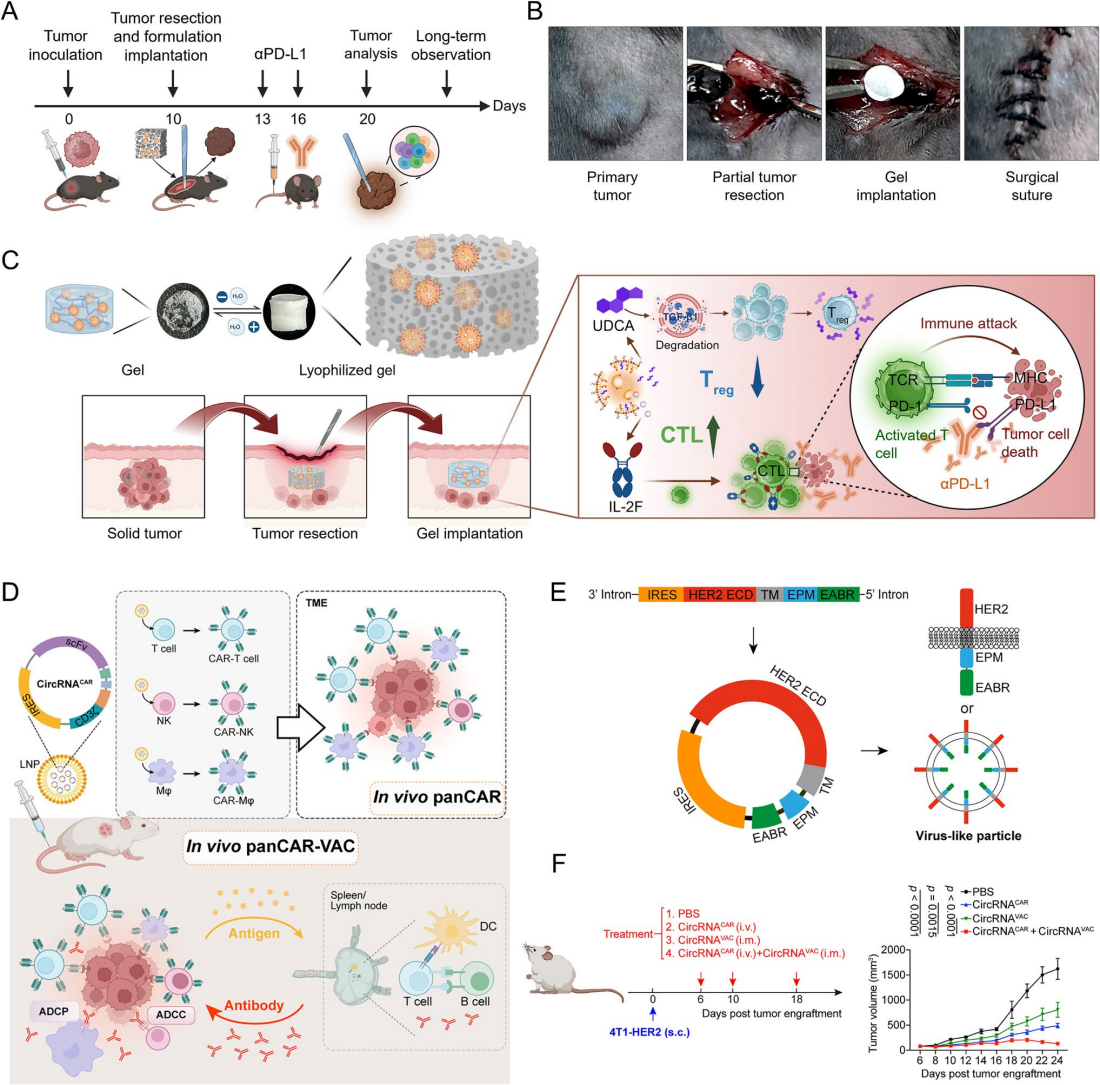
circRNA therapeutics in anti-cancer treatment. **A** The time course of the study shows tumor inoculation, partial resection, hydrogel implantation, αPD-L1 administration, and follow-up analysis. **B** Intraoperative images of primary tumor, partial resection, lyophilized hydrogel implantation, and surgical closure. **C** Mechanistic illustration of the ULNPs-circRNA^IL-2F@LG system combined with αPD-L1, where local gel implantation enables sustained circRNA release, promotes cytotoxic T lymphocyte (CTL) infiltration, reduces Treg activity, and synergistically enhances antitumor immune responses to suppress melanoma recurrence [106]. Reproduced from Kai Yang et al., 2025, Science Advances: under the terms of the Creative Commons CC BY License. **D** Schematic of the in vivo panCAR-VAC approach: circRNA-LNP formulations encoding CAR-related constructs induce antigen presentation and immune activation, driving CAR-T-like activity and enhancing humoral and cellular responses. **E** Design of circRNA vaccines encoding HER2 extracellular domains fused to immune-enhancing motifs (EPM/EABR) to promote assembly of virus-like particles (VLPs), thereby boosting immunogenicity. **F** Experimental setup and tumor growth curves in 4T1-HER2 xenograft models, demonstrating superior antitumor efficacy of circRNA vaccines compared with controls [107]. Reproduced from Yanyan Wang et al., 2025, Cell Reports Medicine: under the terms of the Creative Commons CC BY-NC License

Innovative delivery platforms are further expanding circRNA immunotherapy. A circRNA encoding an IL-2–Fc fusion protein, delivered in ursodeoxycholic acid-based LNPs and combined with a sustained-release hydrogel, markedly prolonged half-life and shifted the CD8^+^/Treg balance toward effective antitumor immunity, producing strong responses in melanoma and glioma models [106]. More recently, circRNAs encoding CAR constructs (circRNACAR) have been used to bypass ex vivo engineering, directly programming T cells, NK cells, and macrophages in vivo into functional “panCAR” effectors. When combined with HER2-targeted circRNA vaccines, this approach produced synergistic tumor control, offering a glimpse of a scalable and less resource-intensive form of cellular immunotherapy [107] (Fig. 4).

### Infectious diseases

Although mRNA vaccines have proved transformative during the pandemic, their broader application is limited by transient expression, inherent instability, and the need for chemical modifications to mitigate innate immune surveillance. CircRNAs, by contrast, lack free 5′ and 3′ termini and therefore resist ribonuclease degradation, supporting more durable protein production in vivo.

**Fig. 5:**
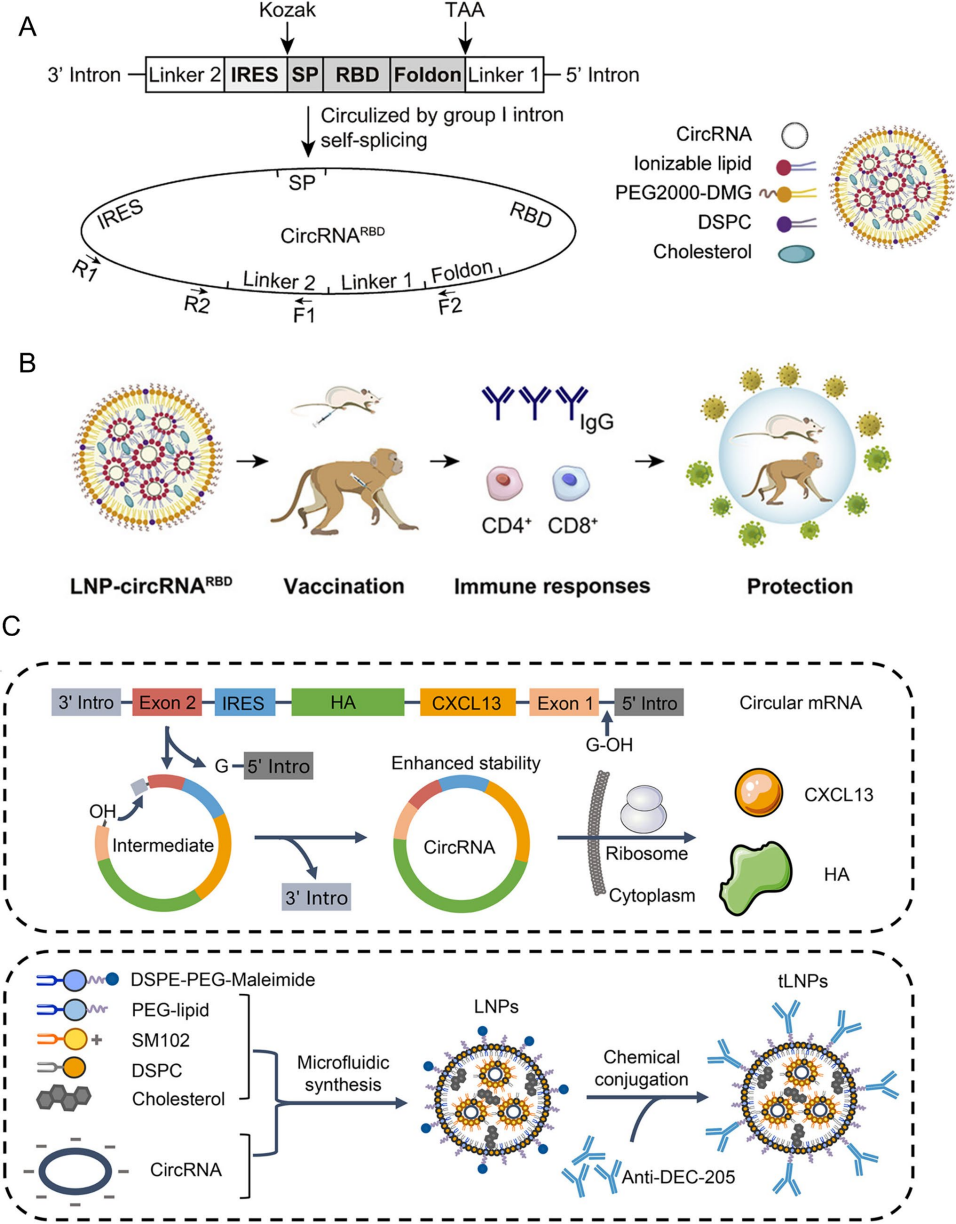
Schematic illustration of circRNA therapeutics in the treatment of infectious diseases. **A** Left: Illustrative schematic of circRNA^RBD^ formation mediated by group I intron self-splicing. SP: signal peptide sequence of human tPA; Foldon: trimerization domain from bacteriophage T4 fibritin. Right: schematic representation of circRNA-loaded LNPs composed of ionizable lipid, PEG2000-DMG, DSPC, and cholesterol. **B** Schematic representation of LNP-circRNA^RBD^ vaccination and induction of immune responses. LNP-circRNA^RBD^ vaccination in mice and nonhuman primates triggers antigen expression, leading to robust CD4^+^ and CD8^+^ T cell responses, IgG production, and protective immunity [108]. Adapted from Liang Qu et al., Cell, 2022, published by Elsevier under the Creative Commons CC BY License. **C** Schematic diagram of circRNA circularization via group I intron autocatalysis and subsequent protein expression. The IRES sequence recruits ribosomes to facilitate cap-independent translation of encoded proteins (e.g., CXCL13, HA). Bottom: workflow for the formulation of targeted LNPs (tLNPs). CircRNAs are synthesized and encapsulated into LNPs (containing DSPE-PEG-Maleimide, PEG-lipid, SM102, DSPC, and cholesterol) using microfluidic synthesis, followed by chemical conjugation with anti-DEC-205 antibodies to generate tLNPs for enhanced delivery67. Reproduced from Jiawu Wan et al., 2024, PNAS: under the terms of the Creative Commons CC BY License

In studies of SARS-CoV-2, a circRNA vaccine designed to express a trimeric spike receptor-binding domain induced robust neutralizing antibody responses as well as cellular immunity in both mice and rhesus macaques. Compared with a 1mΨ-modified mRNA vaccine, the circRNA construct generated higher and more sustained antigen expression and promoted a Th1-skewed response, yielding a higher proportion of neutralizing-to-binding antibodies [108]. Similar advantages have been observed in flavivirus models. A Zika vaccine design based on circRNA encoding a dimeric envelope (E) domain III (EDIII) fused to human IgG1 Fc triggered stronger germinal-center responses and higher neutralizing antibody titers than constructs encoding monomeric or trimeric EDIII, while importantly avoiding dengue antibody-dependent enhancement, supporting circRNA as a safe and effective flavivirus vaccine platform [68]. CircRNAs also enable modular designs that combine antigen and immunomodulators within a single transcript. One example is the co-encoding of CXCL13 alongside viral antigens, delivered in lymph-node-targeted LNPs. This system enhanced neutralizing antibody breadth against influenza and SARS-CoV-2, conferred protection against heterologous influenza challenge in mice, and remained amenable to lyophilization, addressing key barriers to long-term storage and global distribution of RNA vaccines [67] (Fig. 5). Therefore, circRNA vaccines can extend antigen durability, enhance immune quality, and simplify vaccine formulations by integrating adjuvant functions, offering clear advantages over linear mRNA platforms for infectious disease prevention.

### Other diseases

CircRNA therapeutics are also being explored in disease settings beyond infection and cancer, particularly where conventional treatments remain inadequate. Neurological injury is one such case: stroke continues to be a major contributor to persistent neurological impairment, with limited pharmacological options to accelerate recovery. Screening of patient plasma identified circSCMH1 as a protective molecule in ischemic injury. To overcome delivery barriers, researchers engineered EVs functionalized with rabies virus glycoprotein to carry circSCMH1 selectively into the brain. In rodent and non-human primate models, this targeted system improved synaptic remodeling, reduced neuroinflammation, and promoted functional recovery [109].

Chronic wound healing provides another example. Diabetic foot ulcers, which arise from impaired angiogenesis and microvascular damage, are notoriously resistant to treatment. VEGF-A circRNA encapsulated in ionizable LNPs enabled sustained in situ production of VEGF-A, driving angiogenesis and accelerating wound closure in diabetic models. This approach demonstrates how circRNAs can address therapeutic needs unmet by protein or mRNA therapies due to stability and dosing limitations [94].

In vascular disease, aberrant circRNA expression has been linked to maladaptive remodeling. CircMAP3K5, for instance, modulates vascular smooth muscle cell phenotypic regulation through sequestration of miR-22-3p, leading to suppression of TET2. Disruption of the circMAP3K5/miR-22-3p/TET2 signaling pathway has been shown to restrain intimal hyperplasia, indicating a promising therapeutic avenue for restenosis and atherosclerosis. Together, these examples illustrate the expanding scope of circRNA-based interventions in disorders of the nervous, metabolic, and cardiovascular systems [110].

### circRNAs as diagnostic biomarkers

Recent research also highlights circRNAs as promising biomarkers for a variety of diseases due to their high stability and enrichment in biofluids and EVs [111]. In cancer, circRNAs can be detected non-invasively in plasma or urine, helping with early diagnosis, prognosis, and monitoring disease progression [112, 113]. Specific circRNAs correlate with metastasis and poor outcomes in certain cancers, and expression signatures may predict responses to immunotherapies [114]. Beyond cancer, circRNAs are linked to autoimmune and inflammatory diseases, where changes in their levels could aid diagnosis and management [115, 116]. In cardiovascular diseases, some circRNAs modulate key cellular functions and are elevated in conditions like aortic aneurysm, suggesting diagnostic potential [117–119]. Neurological research shows circRNA production is associated with loci for diseases such as Parkinson’s and Alzheimer’s, and their detection in cerebrospinal fluid or blood may enable new diagnostic approaches [120–122]. CircRNAs also show promise as biomarkers in other conditions like gestational disorders [123, 124]. Overall, circRNAs have distinct expression patterns in various diseases and biofluids, supporting their potential as stable, clinically useful biomarkers for diagnosis, prognosis, and treatment response across a broad spectrum of disorders.

## Conclusions and future outlook

Over the past few years, many studies have revealed circRNAs as multifunctional regulators and promising therapeutic agents (Table 1). Due to their closed structure, circRNAs have intrinsic stability, enabling longer persistence in cells than linear RNAs and reducing susceptibility to ribonuclease degradation. These features not only make circRNAs attractive as potential biomarkers and functional modulators but also suggest advantages for their storage and transport compared with conventional RNA formulations. In addition to the natural functions such as miRNA sponges, protein scaffolds, and transcriptional modulators, synthetic circRNAs have been engineered to encode proteins, regulate gene expression, and even guide nucleic acid editing. Advances in high-throughput sequencing continue to unlock novel endogenous circRNAs linked to human disease, while progress in chemical and enzymatic synthesis has improved the generation of protein-coding and non-coding circRNAs for therapeutic testing.

**Table 1:** CircRNA-based therapeutic applications

circRNA	Mechanism	Disease	Biological function	Delivery strategy	Ref.
circRNARBD-Delta	Encoding protein	SARS-CoV-2 infection	Encoding viral antigens to induce adaptive immune responses (antibody production and T cell activation); positive regulator of vaccine stability and immunogenicity	LNP	[108]
circRNA^OVA^	Encoding protein	Cancer vaccine	Stimulating strong antitumor immune responses, enhancing T cell activation; promoting tumor growth inhibition	LNP	[125]
circOVA	Encoding protein	Cancer vaccine	Inducing robust and sustained T cell responses; strengthening cellular immunity	CARTs	[53]
circFAM53B	Encoding protein	Cancer vaccine	Generating cryptic peptides that are presented as neoantigens; eliciting anti-tumor immune responses	Naked circRNA	[48]
GSDMD^ENG^ circRNA	Encoding protein	EIF4G2^+^/PTBP1^+^ tumor	Targeting mitochondrial inner membrane cardiolipin; disrupting tumor cell survival and leading to ablation of EIF4G2^+^/PTBP1^+^ cancer cells	LNP	[64]
circDYM	miRNA sponging	Major depressive disorder	miR-9 sponging; regulator of microglial activation; HSP90 ubiquitination mediator; antidepressant effect	-	[126]
circSCMH1	Binding protein	Acute ischaemic stroke	Neural plasticity enhancer; promoter of functional recovery; EV-deliverable therapeutic	EVs	[109]
circ-SHPRH	Encoding protein	Glioblastoma	Protein-coding circRNA; tumor suppressor; suppressor of glioma tumorigenesis	Plasmid-based overexpression systems	[127]
circLINC-PINT	Encoding protein	Glioblastoma	Tumor suppressor; inhibitor of oncogenic transcriptional elongation	Plasmid-based overexpression systems	[128]
Circ-AKT3	Encoding protein	Glioblastoma	Tumor suppressor; inhibitor of tumorigenicity; PDK1 competitor	Plasmid-based overexpression systems	[129]
circHEATR5B (circ_0054048)	Encoding protein	Glioblastoma	Tumor suppressor; inhibitor of aerobic glycolysis; JMJD5 phosphorylation mediator	Plasmid overexpression & siRNA/shRNA knockdown	[130]
circRNA-5692	miRNA sponging	Hepatocellular carcinoma	Tumor suppressor; miR-328-5p sponging; enhancer of DAB2IP expression; inhibitor of cancer progression	Plasmid-based overexpression systems	[131]
circVAMP3	Binding protein	Hepatocellular carcinoma	Tumor suppressor; CAPRIN1 phase separation driver; c-Myc translation inhibitor	Plasmid-based overexpression systems	[132]
circNFIB (hsa_circ_0086376)	Binding protein	Intrahepatic cholangiocarcinoma	Tumor suppressor; inhibitor of tumor growth; inhibitor of metastasis; MEK1/ERK signaling suppressor	Plasmid-based overexpression systems	[133]
circASH2	Binding protein	Hepatocellular carcinoma	Tumor suppressor; cytoskeleton remodeling mediator, inhibitor of metastasis; phase separation regulator	Plasmid-based overexpression systems	[104]
circZKSCAN1	Encoding protein	Hepatocellular carcinoma	mTOR degradation mediator; inhibitor of HCC progression	Plasmid-based overexpression systems	[134]
circRNA_0001805	miRNA sponging	Nonalcoholic fatty liver disease	miR-106a-5p/miR-320a sponging; ABCA1/CPT1 axis regulator; alleviator of NAFLD	Galactose-modified RBC membrane-coated MOF nanocarrier	[135]
circORC5	miRNA sponging	Gastric cancer	miR-30c-2-3p sponging; AKT1S1 axis regulator; inhibitor of cancer progression	Plasmid-based overexpression systems	[136]
circDIDO1	miRNA sponging	Gastric cancer	miR-1307-3p sponging; SOCS2 axis regulator; inhibitor of cancer progression	RGD-modified exosomes	[137]
circURI1	Binding protein	Gastric cancer	Tumor suppressor; hnRNPM interactor; alternative splicing modulator; metastasis inhibitor	Plasmid-based overexpression systems	[138]
circMAPK1	Encoding protein	Gastric cancer	Tumor suppressor; MAPK signaling suppressor; inhibitor of cancer progression	Plasmid-based overexpression systems	[139]
circDIDO1	Binding protein/Encoding protein	Gastric cancer	Tumor suppressor; DIDO1-529aa protein encoder; PRDX2 stability regulator; inhibitor of cancer progression	Plasmid-based overexpression systems	[140]
circFNDC3B	Encoding protein	Colon cancer	Tumor suppressor; inhibitor of tumor progression and EMT	Plasmid-based overexpression systems	[141]
circPLCE1	Encoding protein	Colorectal carcinoma	Tumor suppressor; NF-κB regulator; RPS3 ubiquitin-dependent degradation promoter; inhibitor of cancer progression	Plasmid-based overexpression systems	[142]
circFndc3b	Binding protein	Myocardial infarction	Cardiac repair modulator, FUS interactor; VEGF-A axis regulator; promoter of cardiac regeneration	Plasmid/viral vector delivery in mice	[143]
circPTPRA	miRNA sponging	Non-small-cell lung carcinomas	EMT inhibitor; metastasis inhibitor; miR-96-5p sponging	Plasmid-based overexpression systems	[144]
circSLC8A1	miRNA sponging	Bladder cancer	Tumor suppressor; miR-130b/miR-494 sponging; PTEN regulator; inhibitor of cancer progression	Plasmid-based overexpression systems	[145]
circCAR-Vac	Encoding protein	Cancer	Enhancing anti-tumor immunity by stimulating T cell activity and synergistically boosting the efficacy of in vivo CAR T cell therapy	LNP	[107]
IL-12 circRNA	Encoding protein	NSCLC	Enhancing anti-tumor immunity and potentiating lung cancer immunotherapy	LNP	[92]

Compared with vaccines based on pathogens, DNA, or linear RNA, circRNAs offer distinct pharmacological advantages. They avoid risks of genomic integration and associated mutational risks, and can achieve durable expression even without extensive nucleotide modification. Nonetheless, clinical translation remains at an early stage, as no circRNA therapeutics have reached regulatory approval. Major obstacles include the efficient circularization of long in vitro-transcribed precursors, the removal of linear byproducts during purification, and the optimization of circRNA translation to levels comparable with mRNA [146]. Delivery remains another challenge. For example, ionizable LNPs are currently the most advanced carriers, but they exhibit biases toward certain tissues such as the liver and still face limited endosomal escape. Moreover, interactions among lipid components, including sterols and helper lipids, complicate formulation optimization, suggesting that combinatorial or machine learning-guided design may be necessary for LNP formulation [91, 147].

Continued development in this field will depend on overcoming several critical obstacles, including the improvement of large-scale circularization and purification, robust delivery systems that can target diverse tissues, and therapeutic efficacy in vivo. Computational tools, from in silico ribozyme design to AI-guided optimization of translation elements, are likely to accelerate this progress. At the manufacturing level, establishing GMP-compliant workflows for circRNA synthesis and scalable nanoparticle formulations will be critical. Promising innovations such as self-amplifying circRNA constructs or replicon-inspired designs may further extend durability, reducing dosing requirements.

Although circRNAs were barely recognized a decade ago, they are now emerging as promising candidates for next-generation RNA therapeutics. With coordinated progress in chemistry, engineering, and clinical evaluation, circRNAs hold the potential to become the first wave of RNA therapeutics beyond mRNA, expanding RNA therapeutics beyond vaccines to a wider range of diseases.

## Data Availability

Data sharing is not applicable to this article, as no new data were created or analyzed in this study.
